# Toll-like Receptors in Chronic Lymphocytic Leukemia

**DOI:** 10.4084/MJHID.2012.055

**Published:** 2012-08-09

**Authors:** Marta Muzio, Eleonora Fonte, Federico Caligaris-Cappio

**Affiliations:** 1 Division of Molecular Oncology, Ospedale San Raffaele, Milano, Italy; 2 Università degli Studi di Pavia, Pavia, Italy; 3 Università Vita-Salute San Raffaele, Milano, Italy; 4 Department of OncoHematology, Ospedale San Raffaele, Milano, Italy

## Abstract

Toll-like receptors belong to the pattern recognition receptors family present on a variety of immune cells including normal and malignant B-cells. They act as immediate molecular sentinels of innate immunity but also act as a molecular bridge between the innate and the adaptive immune response; distinct Toll-like receptors are able to bind specific pattern molecules of bacteria, viruses and autoantigens. In this review we will briefly introduce the Toll-like receptor family and their expression pattern, signaling and function in the B cell context; following we will summarize the published data on TLR in chronic lymphocytic leukemia, and we will discuss their emerging role in the modulation of leukemia pathobiology.

## Introduction

Inflammation primarily defends the host organism against infections and is a self limiting process; however, its deregulation leads to chronic inflammatory processes that may favor the development of cancer.^1^ Toll-like receptors (TLR) are well known for their key role regulating innate immunity; emerging evidence support their key regulatory role also in tumor biology. Among other microenvironmental elements, TLR may also play a role in hematologic malignancies, especially in chronic lymphoid malignancies.^2^,^3^ First, we will briefly introduce the role of TLR in normal immune cells. Next we will describe available data on the expression and function of TLR in malignant B lymphocytes. On these bases we will discuss the pathobiology of TLR in Chronic Lymphocytic Leukemia (CLL).

## Toll-like Receptors (TLR)

**Figure 1:**
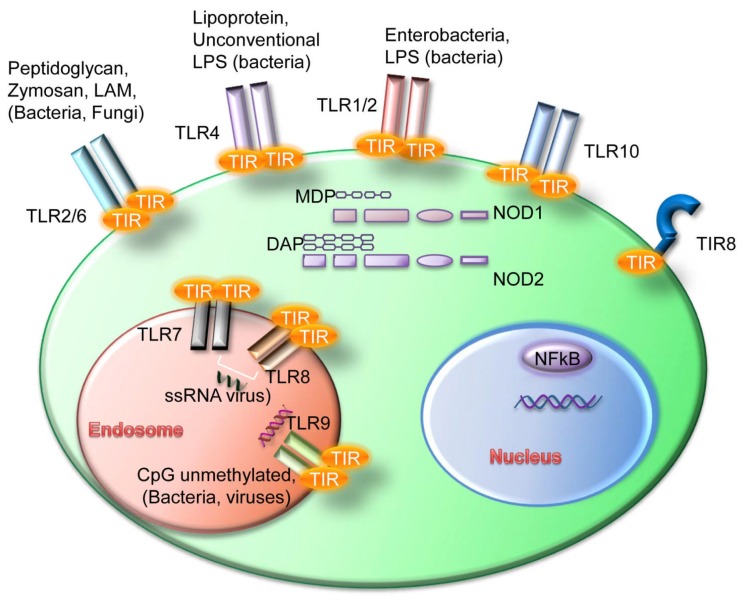
Schematic representation of the TLR expression pattern in CLL cells. MDP: muarmyldipeptide; DAP: D-glutamyl-diaminopimelic acid; ssRNA: single strand RNA.

Innate immune cells express various pattern-recognition receptors (PRR) which recognize common signatures of molecules that are important components of bacteria and viruses and are called pathogen-associated molecular patterns (PAMP).^4^ The response of the innate immune system plays a central role not only in eliminating infectious agents but also in developing pathogen-specific adaptive immunity mediated by B and T cells. Toll-like receptors (TLR) are PRR expressed by a variety of leukocytes as well as by non-immune cells present in particular sites of barrier function such as intestinal or airway epithelia. However, it has been demonstrated that they are activated not only by exogenous PAMP but also by endogenous ligands, so called “danger signals”. These danger-associated molecular patterns (DAMPs) are host-derived TLR ligands^5^,^6^ (see Figure 1 for a schematic representation of TLR and ligands).

TLR are grouped into the same family based on their sequence similarity and structural features; there are ten TLR in humans and twelve in mice where TLR10 pseudogene does not translate into a functional protein.^4^ They are expressed within distinct cellular compartments: TLR1, TLR2, TLR4, TLR5, TLR6, and TLR10 are present on the cell surface whereas TLR3, TLR7, TLR8 and TLR9 are localized into intracellular vesicles such as endosomes, lysosomes and ER. Intracellular TLR are transported into the vesicles via the transmembrane protein UNC93B1 which is localized in the ER of the cells.^7^ Each TLR can sense specific PAMPs; in details, TLR1 can form heterodimers with TLR2 and bind tri-acetylated lipopeptides, which are coupled with peptidoglycan layer of bacteria. Another well characterized heterodimer is TLR2/TLR6 which recognizes di-acetylated lipopeptides and bacterial cell wall components such as lipoteichoic acid or peptidoglycans, mycobacterial cell wall components like lipomannans and the yeast cell wall zymosan. TLR3 binds to double stranded RNA from viral sources while TLR4 responds to lipopolysaccharide (LPS) mostly from Gram-negative bacteria; the only known ligand for TLR5 is flagellin. The intracellular TLR7 and TLR8 share the same ligand, the single stranded RNA from viruses, while TLR9 binds to DNA-containing unmethylated CpG motifs which are commonly found in bacterial DNA.^4^,^8^

Distinct TLR can also sense different DAMPs.^5^ TLR2 and TLR4 are the best characterized in this respect; they have several endogenous ligands such as heat shock proteins including HSP70 and Gp96),^9^,^10^ HMGB1,^11^ extracellular matrix (ECM) molecules^12^ and their fragments.^13^ TLR1/TLR2 were shown to be activated by β-defensin-3;^14^ TLR3 by self-nucleic acids.^15^ TLR7 and TLR9 can also participate to autoantigen response together with the B cell receptor by recognizing RNA-associated autoantigens^16^ and chromatin-IgG complexes^17^ respectively.

The last discovered TLR10 is an orphan receptors; however, sequence analysis as well as chimeric receptors experiments suggested that human TLR10 and TLR1 share common mechanisms of innate immune sensing but not signaling.^18^,^19^

CD180 (also named RP105 or Ly64) is homologous to TLR4 but lacks the intracellular TLR-like domain.^20^ RP105 is associated with MD-1 which is indispensable for its cell-surface expression.^20^ RP105-deficient B cells are defective in response to TLR2 and TLR4 ligands.^21^ However, it was also demonstrated that RP105 may prevent the interaction of LPS with TLR4 in macrophages and dendritic cells^22^ suggesting different roles of RP105 within differet cell types. Other studies demonstrated that CD180 has a role in B-cell activation (by up-regulating CD86) and proliferation.^23^ Furthermore CD180 stimulation induces B cell proliferation and differentiation, causing increases in IgG, and integrates MyD88-dependent TLR signals to modulate proliferation, production of cytokines, and differentiation.^24^

## NOD-like Receptors

Another family of PRRs, the NOD-like receptors family (NLRs), is composed of several cytosolic molecules including the first discovered NOD1 and NOD2 (also known as CARD4 and CARD15). NLRs show a variable modular structure and contain different domains: a CARD or pyrin domain at the N-terminal portion; a central nucleotide binding and oligomerization domain; Leucine Rich Repeats (LRR) at the C-terminal domain. While NODs contain a CARD domain, NALPs contain a pyrin domain NAIP being an exception as it contains different BIR domains.^25^ NOD1 and NOD2 are well characterized intracellular molecules that recognize bacterial peptidoglycans. Their expression is wide and includes B-cells where a synergism between NOD-like receptors and Toll-like receptors was observed.^26^ NOD1 binds iE-DAP dipeptide which is found in peptidoglycan of most Gram-negative bacteria while NOD2 responds to MDP (muarmyldipeptide) which is the minimal bioactive peptidoglycan motif common to all bacteria. NOD stimulation leads to inflammatory genes transcription through NF-kB and MAPK activation.^27^,^28^

## TLR Signaling

**Figure 2:**
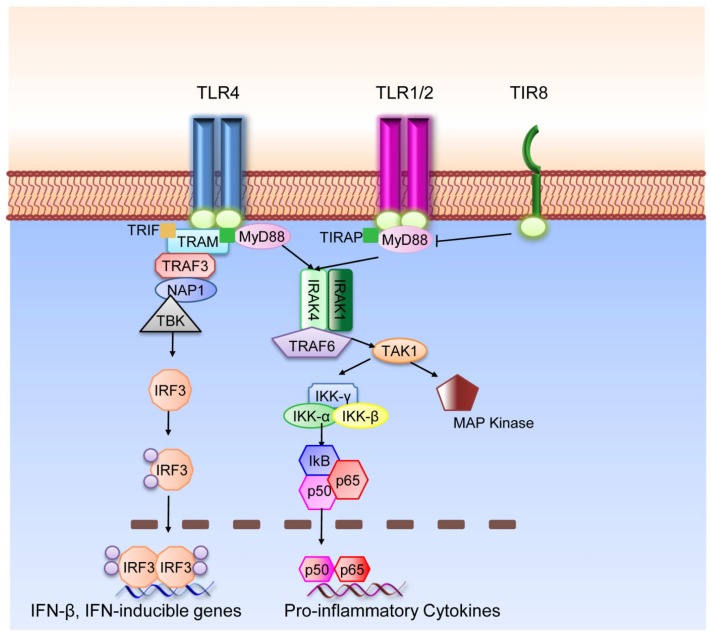
Schematic representation of TLR signialing pathway.

TLR are type I integral membrane glycoproteins and have a modular structure. The extracellular N-Terminal domain consists of approximately 16–28 Leucin-Rich Repeats (LRRs) which mediate ligand binding specificity. The cytoplasmic domain is highly conserved and termed Toll-IL-1R (TIR) domain according to the high similarity shared with the Drosophila Toll and the mammalian IL-1R protein.^29^ This domain acts as binding site for downstream adaptor molecules that mediate the signal to others proteins (see Figure 2 for a schematic representation of TLR signaling pathways). Two main adaptors are recruited to the TIR domain; MyD88, which is recruited to the TLR-TIR domain together with Mal (MyD88 adaptor-like) also called TIRAP (TIR-domain-containing adaptor protein), and TRIF (Toll-receptor-associated activator of interferon).^30^ Throughout the first pathway, the induction of specific gene expression is mediated by the NF-κB (nuclear factor k B) transcription factor, AP-1 (activating protein 1) or IRF1, 5 and 7 (interferon-response factor); on the other hand, the second pathway is regulated by IRF3 and NF-κB.^31^ Only TLR4 can trigger downstream signals through both pathways; all the others, except for TLR3, act via MyD88. The death domain of MyD88 recruits IRAK family members to the TLR signaling complex, activate them and transmit the signal to TRAF6 which allows the phosphorylation of IKK. The pathway flows with the activation of NF-κB and the recruitment of TAK1 that induces the MAPK pathways. These signaling cascades eventually induce the transcription of inflammatory cytokines, type I or II interferons and chemokines.^30^

Fine tuning of TLR and IL-1R family (ILR) is regulated by the inhibitory receptor TIR8 (Toll IL-1R 8), also known as Single Ig IL-1 related receptor (SIGIRR); TIR8 acts as a decoy target for TLR and ILR (IL-1R, IL-18R, IL-33R/ST2) signaling molecules.^32^,^33^ SIGIRR inhibits interleukin-1 receptor-and TLR-mediated signaling through different mechanisms. Both extracellular domain and the intracellular portion of TIR8 are involved; the intracellular TIR domain of TIR8 sequesters MyD88 and IRAK-1, while the extracellular domain interferes with heterodimerization of IL-1R1 and IL-1AcP.^34^,^35^

Other signaling molecules such as IRAK-M and SOCS-1 negatively regulate IRAK. IRAK-M expression has been shown to be restricted to monocytes and macrophages; it blocks the dissociation of IRAK1/4 and the following activation of TRAF6.^36^ SOCS-1 knock-out mice show over-expression of different cytokines specifically after treatment with LPS.^37^

## TLR in Normal B Lymphocytes

The TLR expression pattern is quite specific and unique for each cell type;^38^ in normal human B-cells TLR1, TLR2, TLR6, TLR7, TLR8, TLR9 and TLR10 are prevalently expressed; in contrast TLR repertoire in mouse B cells includes high levels of TLR4 and no TLR10 protein.^39^–^41^ In human cells, TLR expression is rapidly up-regulated by BCR triggering of naïve B cells suggesting a synergism between BCR and TLR leading to B-cell proliferation and differentiation.^39^,^40^ TLR expression is also specific for each B-cell subset.^42^ B cells of the inflamed tonsils show abundant TLR expression.^43^

In general terms TLR stimulation can trigger activation, proliferation and differentiation of B cells; nevertheless, TLR ligation with specific ligands can induce specific responses in the B cell subsets analyzed either in mouse models or human system (see specific reviews on this topic).^44^–^46^ In human cells It was shown that maintenance of serological memory can be achieved by polyclonal activation, and memory B cells can be activated by CpG and cytokines without need for BCR triggering.^47^ However, it has been proposed a model in which the costimulation of three different signals derived from BCR, CD40 and TLR is required to induce full activation, proliferation and differentiation of naive B-cells.^48^ Recent studies also showed that a specific culture system using CpG together with sequential steps for T-cell-independent activation of naive human B cells can also induce plasma-cell differentiation.^49^

## Expression Pattern of TLR in CLL Cells

Already before the discovery of TLR9 as the cognate receptor for unmethylated-CpG-oligonucleotides,^50^ these immunostimulatory agents have been used to stimulate leukemic CLL cells for immunotherapeutic strategies.^51^ Therefore, it was assumed, and soon confirmed, that CLL cells express functional TLR9, similar to normal B lymphocytes.^52^,^53^ However, only recently the full expression pattern of TLR was assessed in CLL samples by different groups.^54^–^56^

The expression of TLR in CLL is quite heterogeneous between patients but most cases express TLR1, TLR2, TLR6, TLR10 on the cell surface, and TLR7, TLR8, TLR9 within endosomes^55^ thus resembling normal mature B lymphocytes.^39^,^40^,^43^ Recently, we have studied the full expression profile of mRNA for TLR and signaling molecules in a large group of chronic lymphocytic leukemia (CLL) patients to search for potential differences in specific subsets of patients. At cohort levels, CLL cells show high expression of TLR7, intermediate expression of TLR1, TLR6, TLR10 and low expression of TLR2, TLR4, TLR8 and TLR9. As for TLR4 and TLR8 a significant variation was observed among different samples.^57^ Comparison in subgroups of cases carrying mutated or unmutated IGHV genes revealed few significant differences in TLR signaling molecules; up-regulation of TLR8 mRNA and down-regulation of TLR4 were observed in the unmutated subgroup.^57^

The TLR related molecule RP105, also called CD180 or Ly64 was shown to be variably expressed by leukemic cells.^58^,^59^ Significantly higher levels of CD180 were expressed by CLL cells with mutated IGVH genes as compared to unmutated CLL.^58^

Further, expression of NOD1 (CARD4) and NOD2 (CARD15) mRNA was studied in a group of CLL patients in comparison with MEC1 cell line and normal B-lymphocytes. Both NOD molecules, including two different isoforms of NOD1, were expressed in all B-cell types analyzed.^55^

We have previously shown that both normal and leukemic B cells express detectable levels of TIR8 mRNA;^55^ however, by PCR array analysis, malignant B cells appear to have very low levels of mRNA.^60^ Since mRNA and protein levels of TIR8 have been shown to be differentially regulated,^61^ it will be important to analyze TIR8 protein expression on the cell surface of normal and leukemic B lymphocytes.

Finally, our group recently analyzed the mRNA expression pattern of the molecules regulating Toll-like receptor signaling pathway in a large cohort of CLL patients; different members of the NFKB, JNK/p38, NF/IL6 and IRF pathways are intermediately-to-highly expressed, while inhibitors of TLR activity are generally low-to-undetectable, indicating that the TLR signaling framework is competent in CLL cells.^57^

To note, all these studies analyzed leukemic cells isolated from the peripheral blood of patients; it will be of interest to compare TLR expression pattern within lymphoid tissues where one would expect TLR ligation to occur. In fact, it was reported that, in addition to BCR, TLR signaling pathways could contribute to NF-kB activation specifically in the lymph node microenvironment.^62^

## Gene Polymorphisms and Mutations of the TLR Pathway in CLL

Functional polymorphisms in TLR genes were analyzed by different groups to determine if they influenced lymphoma susceptibility. TLR6 variants were found to be important in different B cell lymphomas including CLL;^63^ the TLR2-16933T>A variant was associated with a decreased risk of CLL;^64^ two TLR10-TLR1-TLR6 variants in moderate linkage disequilibrium were significantly associated with Non Hodgkin Lymphoma including CLL cases.^65^

More recently, MyD88 oncogenic mutations were described in different B cell malignancies; in details, recurrent single point mutations were found in 29% of ABC type Diffuse Large B Cell Lymphoma cases,^66^ 13% of Splenic Marginal Zone Lymphoma cases,^67^ 36% of Primary Central Nervous System Lymphoma cases,^68^ and 3–10% of CLL cases.^69^–^71^ Since MyD88 is a signaling molecule specific for TLR and IL-1R family, this mutation may affect specific signaling pathways in leukemic cells which may be considered as novel therapeutic targets. Indeed, inhibition of the MyD88 downstream kinases IRAK1/4 with small molecule inhibitors was shown to effectively block TLR signaling cascade *in vitro* and to induce cell death of lymphoma cells bearing specific MyD88 mutations.^66^

## TLR and Activation of Leukemic Cells

Several reports (mainly focused onto TLR9) showed that CpG immunostimulatory oligonucleotides shape an immunogenic phenotype in CLL cells.^51^,^72^ Several surface antigens have been investigated in CLL cells before and after CpG addition to the cell culture; among these CD25, CD40, CD54, CD80, CD86 CD95, MHCI, MHCII;^51^,^73^ the expression of these molecules contribute to increase of the immunogenicity of the leukemic cells that are *per se* weakly immunogenic and may escape the control exerted by tumor-reactive T cells.^74^–^76^ Stimulation of CLL cells with different agonists of TLR also increases the number of CD25, CD80 and CD86 positive cells, and this was demonstrated for PAM3CSK4 (palmitoyl-3-cysteine-serine-lysine-4 binding to TLR1/2 heterodimer), MALP-2 (Mycoplasmal Macrophage-activating Lipopeptide-2 binding to TLR2/6 heterodimer) and MDP (Muramildipeptide binding to NOD2).^56^,^77^ TLR7 stimulation is also able to increase the expression of costimulatory molecules on leukemic cells (CD25, CD80, CD86) and the production of inflammatory cytokines including TNFα and IL-6.^78^,^79^ Moreover, the TLR9 ligand CpG induces the production of TNFα, IL-10 and to a lesser extent of IL6.^51^,^56^,^73^,^80^ Given the ability of CpG to induce CD25 expression, TLR9 ligands were also tested *in vitro* in combination with a specific anti-CD25 immunotoxin to treat CLL cells.^81^ Both TLR7 and TLR9 agonists have been studied for immunotherapy approaches in preclinical models of CLL *in vitro*,^73^,^82^,^83^ and are currently under clinical investigation^84^–^86^ (see reference^87^ for a recent review on this topic).

More recently, our group showed differences in the induction of co-stimulatory molecules and/or apoptosis in mutated vs. unmutated CLL. Different responses were also identified in subsets with stereotyped BCR.^79^ The distinct patterns of TLR/NOD2 functional activity in cells from CLL subgroups defined by the molecular features of the BCR might prove relevant for elucidating the immune mechanisms underlying the natural history of CLL and for defining subgroups of patients who might benefit from treatment with specific TLR ligands.

## TLR, Proliferation and Apoptosis of Leukemic Cells

Previous reports showed that CpG immunostimulatory oligonucleotides induce proliferation of CLL cells, either alone or in combination with IL-2.^51^,^72^ However, it was also reported that TLR9 signaling by CpG-B oligonucleotides may induce an apoptotic pathway in CLL.^56^,^73^ Further, several groups described a heterogeneous response to TLR9 stimulation in terms of proliferation and apoptosis within different groups of patients;^56^,^88^ in details, it was reported that CpG induced apoptosis in mutated, and proliferation in unmutated patient samples.^53^,^89^ It is relevant to note in this context that phosphorothioate oligonucleotides may induce apoptosis of CLL cells independently of their CpG motifs, indicating that the presence of a chemical backbone and nucleotide composition may contribute to the observed cell death.^90^

As for additional TLR, we previously showed that TLR1/2 and TLR2/6 heterodimers can activate and protect leukemic cells from spontaneous apoptosis *in vitro*.^55^,^79^ Again, different groups showed heterogeneity among patients samples and apoptosis induction was reported in a proportion of cases;^56^ nevertheless, it was recently shown that distinct innate immunity pathways can be activated in subgroups of CLL with distinct immunoglobulin receptors.^79^

## Dissecting the Role of TLR in CLL Pathobiology

CLL patients are often associated with an increased frequency and severity of infections which is a characteristic feature of the disease.^91^ In addition, autoimmune complications can occur in up to a quarter of CLL patients.^92^ It was also suggested that common infections may play a role in CLL etiology;^93^–^95^ this may be due to underlying immune disturbance in CLL patients, and/or to a direct effect of microbial antigens on the leukemic clone. Given all this, one could hypothesize that inflammation or autoimmunity mediated by distinct TLR may also play a role in regulating the development, progression and/or accumulation of CLL. Indeed, in mouse models of CLL the lack of the inhibitory receptor TIR8, which allows an unabated TLR-mediated stimulation, triggers leukemia progression *in vivo*.^96^ However, these findings do not allow to understand whether TLR contribute (and to what extent) to early or late or both/any phase of disease progression.

Since TLR can improve immune response but may also be involved in modulating tumor cell proliferation and apoptosis, the possibility that TLR activity may shuttle between defense from and promotion of leukemic growth has to be taken into account. Future studies combining *in vitro* and *in vivo* approaches will help to identify the specific role of TLR within specific subsets of patients. Finally, kinetic studies will help elucidating the distinct role of distinct TLR in different phases of disease initiation, accumulation and/or progression.
